# Acceptability, feasibility, and impact of the MyGut digital health platform in the monitoring and management of inflammatory bowel disease

**DOI:** 10.1093/jcag/gwae029

**Published:** 2024-09-06

**Authors:** Jamie Zhen, Maude Simoneau, Pooja Sharma, Pascale Germain, Pascale Watier-Levesque, Abdulrahman Othman, John K Marshall, Waqqas Afif, Neeraj Narula

**Affiliations:** Department of Medicine, University of Ottawa, Ottawa, ON K1H 8L6, Canada; Faculty of Medicine, McGill University, Montreal, QC H3G 2M1, Canada; Institute for Management and Innovation, University of Toronto, Toronto, ON L5L 1C6, Canada; Research Institute of the McGill University Health Centre, Montreal, QC H3H 2R9, Canada; Research Institute of the McGill University Health Centre, Montreal, QC H3H 2R9, Canada; Research Institute of the McGill University Health Centre, Montreal, QC H3H 2R9, Canada; Department of Medicine (Division of Gastroenterology), McMaster University, Hamilton, ON L8S 4K1, Canada; Farncombe Family Digestive Health Research Institute, McMaster University, Hamilton, ON L8S 4K1, Canada; Department of Gastroenterology, McGill University, Montreal, QC H3G 2M1, Canada; Department of Medicine (Division of Gastroenterology), McMaster University, Hamilton, ON L8S 4K1, Canada; Farncombe Family Digestive Health Research Institute, McMaster University, Hamilton, ON L8S 4K1, Canada

**Keywords:** inflammatory bowel disease, IBD, digital health monitoring, digital health intervention, health monitoring platform, eHealth, telemedicine, acceptability, feasibility, MyGut

## Abstract

**Background:**

Digital health monitoring may help facilitate self-management strategies when caring for patients with inflammatory bowel disease (IBD).

**Aims:**

This study investigated the feasibility of implementing the MyGut health application when caring for patients with IBD and evaluated whether its use improved health outcomes.

**Methods:**

We conducted a prospective trial in 2 Canadian hospitals from 2020 to 2023. Patients with IBD were recruited from gastroenterology clinics, and the MyGut application was installed onto their mobile devices. Metrics such as acceptability, satisfaction, feasibility, quality-of-life scores (measured through the short IBD questionnaire [SIBDQ]), and resource utilization were collected throughout the 1-year follow-up period.

**Results:**

Of the 84 patients enrolled, 58 patients (69%) continued to use the app until the study completion. At recruitment, all 84 patients (100%) were willing to use the MyGut application after a brief tutorial. There was a significant improvement in the SIBDQ scores after 1 year of MyGut use (mean = 56.0, SD 8.85 vs 52.0, SD 9.84) (*P* = .012). However, only 42.9% (21/49) of the patients were willing to continue using the application after 1 year, a significant decrease compared with the 71.4% (35/49) who were willing to continue after 2 months (*P* = .001). No differences were observed in the number of emergency room visits/hospitalizations (*P* = .78) before and after 1 year of MyGut use.

**Conclusions:**

This study demonstrates that patients are willing to use digital health monitoring platforms and this may lead to improved quality of life. However, sustained efforts must be made to optimize its long-term feasibility.

## Introduction

Inflammatory bowel disease (IBD) is a chronic, inflammatory, gastrointestinal disorder. Patients with IBD commonly suffer from symptoms such as fatigue, weight loss, abdominal pain, diarrhoea, and bloody stools. Consequently, studies have found that patients with IBD have elevated rates of depression/anxiety, lower quality of life, increased healthcare utilization, and diminished productivity and social isolation.^1–3^ As a chronic, lifelong condition, the management of IBD largely focuses on the induction and maintenance of remission, with surgery reserved for refractory cases.

Similar to diabetes and rheumatoid arthritis, proactive care through “treat-to-target” strategies is now considered best practice in IBD management.^4^ These strategies target a tight state of remission with no disease activity. However, this requires frequent monitoring of validated disease activity measures.^5^ Despite these evidence-based recommendations, the practical application of these strategies is often limited by time and resource constraints. Accordingly, office visits can be infrequent and management decisions are often reactive instead of proactive.^6^ Digital health monitoring platforms can offer a potential solution to overcome these limitations by providing patients with a more proactive means to monitoring, encouraging effective self-management strategies, and complimenting traditional office-based encounters.

With increased access to technology, patients have the means to actively participate in their care and enhance the overall healthcare efficiency. Digital health technologies can be used to facilitate health-related communication between patients and healthcare providers, provide patients with the tools to track their medical data, and help disseminate valuable health information. Furthermore, studies have found that those who use digital health applications feel more empowered, more engaged, and more connected with their healthcare team.^7^

While health monitoring platforms have been established for various chronic conditions, including asthma, diabetes mellitus, and depression, those tailored specifically for IBD remain relatively limited.^8^ However, some have shown decreases in healthcare utilization and improvements in quality of life, symptom management, medication adherence, and IBD-related knowledge.^9^ Nevertheless, the certainty of the evidence remains low, and more studies investigating the impact of health monitoring platforms in IBD, particularly those that integrate healthcare professionals into patient care, are necessary to validate the benefits of these interventions.

The objective of this study was to investigate patients’ acceptance of the IBD health monitoring platform, MyGut, to evaluate whether its use leads to better quality of life/improved health outcomes and to determine the feasibility of its long-term use.

## Methods

### MyGut health monitoring platform

**Figure 1.**
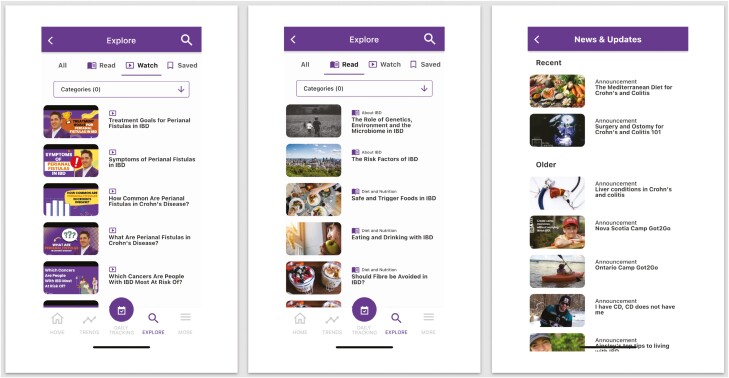
Educational material within the MyGut patient application.

**Figure 2.**
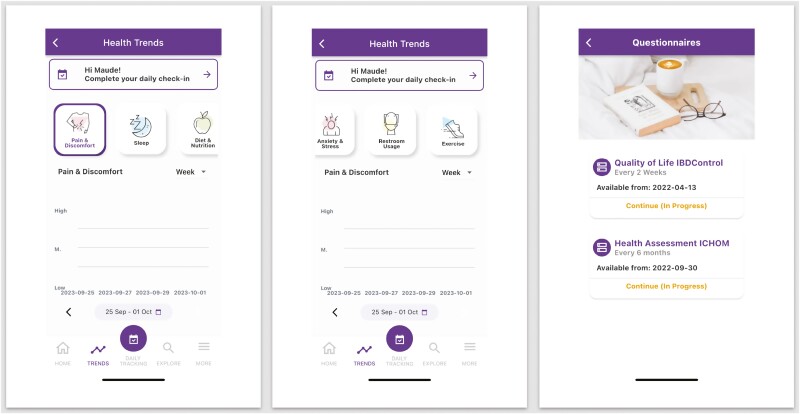
Health-tracking questionnaires within the MyGut patient application.

**Figure 3.**
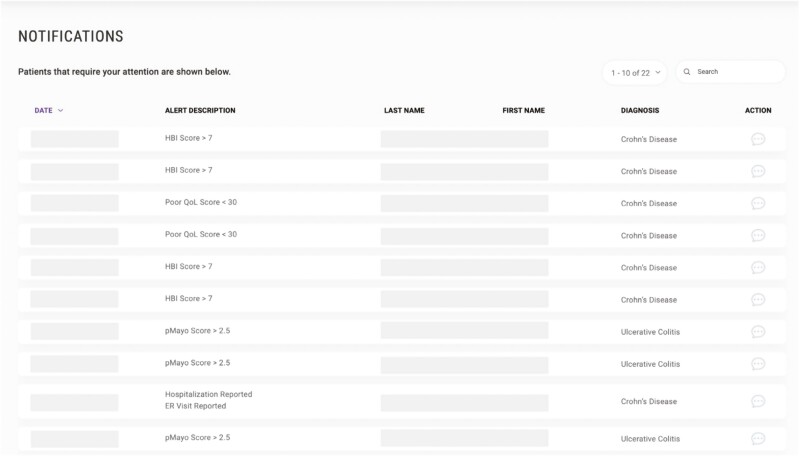
Notification centre within the MyGut physician dashboard.

MyGut is a cloud-based patient-reported outcome electronic application developed by Crohn’s and Colitis Canada in partnership with a consortium of physicians, researchers, and patient advocates. Specifically, MyGut was tailored to the specific needs of Canadians with IBD and healthcare practitioners through a consensus-driven approach. Through the application, patients can access a broad array of IBD-specific educational material (Figure 1). Furthermore, patients can track their symptoms, quality-of-life metrics, and frequency of emergency room (ER) visits/IBD-related hospitalizations (Figure 2). Their IBD team can access these data, allowing for the opportunity to provide timely management suggestions including lifestyle modifications, optimizing treatment regimes, or expediting clinic visits. Furthermore, the physician dashboard permits the flagging of patients with suboptimal disease control who may require further attention (Figure 3).

### Study protocol

We conducted a multi-centre, single-arm trial at McMaster University (Hamilton, Ontario) and McGill University (Montreal, Quebec), among their affiliated hospitals, from September 2020 to September 2023.

Patients with IBD were recruited from gastroenterology clinics during their routine office visits. Their details were sent to the MyGut research team for further information before being asked to install the MyGut application onto their mobile devices. Eligibility criteria included subjects aged 18 years or older with an established diagnosis of IBD, who have access to a MyGut compatible device (mobile phone, tablet, computer, etc.), and who have the ability to use the MyGut application (currently available only in English). Exclusion criteria included the presence of any condition that, in the opinion of the investigators, may make it difficult to use the MyGut application, such as cognitive impairment. There were no incentives provided for participation.

Once enrolled, the patients completed a set of baseline questionnaires investigating their willingness to use the application, satisfaction with care, symptoms as measured by PRO-2 instruments (abdominal pain and stool frequency for Crohn’s disease, stool frequency, and rectal bleeding for ulcerative colitis), quality-of-life scores (measured through the Short Inflammatory Bowel Disease Questionnaire [SIBDQ]), and resource utilization (self-reported ER visits or hospitalizations). The patients were also asked to complete PRO-2 questionnaires and the SIBDQ within the MyGut application every 2 weeks. Their healthcare team routinely checked the provider portal every 2 weeks, and the patients were automatically flagged if they were found to have SIBDQ scores of <30. Subsequent management decisions were taken based on physician discretion. After 2 months of using the application, feasibility questionnaires were completed. At the end of the 1-year study period, the patients completed the end-of-study questionnaires.

### Study instruments

Various symptom score indices/questionnaires were used to evaluate the patients throughout the study.

### Acceptability questionnaire

To determine the willingness of the patients to incorporate the MyGut application into their care, an acceptability questionnaire was administered at the start of the study. The questionnaire assessed whether the patients were open to using the application following a brief tutorial. The tutorial ensured that the patients were informed about the content available in the application and how it was intended to complement their care.

### Feasibility questionnaire

A feasibility questionnaire was adapted from the Telehealth Usability Questionnaire, a validated tool used to evaluate the usability of both telehealth services and mobile health tools.^10–12^ This questionnaire encompassed 6 domains designed to assess patient perceptions regarding the usefulness, ease of use, effectiveness, reliability, and satisfaction with a particular telehealth service. Some statements in the original questionnaire were combined for brevity and ease of use. This survey was assessed for face validity by the authors with experience in digital health. The patients were asked to complete this questionnaire after using the MyGut application for 8 weeks and 1 year, respectively.

### Symptom control and quality-of-life questionnaire for IBD

The SIBDQ is a validated tool for the measurement of health-related quality of life in adult patients with IBD.^13^ This questionnaire assesses 4 domains, including bowel symptoms, emotional health, systemic systems, and social functioning. Integrated into the MyGut application, the patients were asked to complete the SIBDQ at baseline, every 2 weeks, and at the end of the study at 1 year. On the basis of the responses, clinicians can flag patients with suboptimal scores who might require intervention.

### Patient satisfaction with care

The Patient Activation Measure-13 (PAM-13) is a satisfaction questionnaire previously validated for chronic disease management and was adapted for this study.^14^ This questionnaire evaluates patient knowledge, skills, and confidence in managing their condition. For brevity and ease of use, various questions were removed from the original questionnaire. The patients completed this questionnaire at baseline and at 52 weeks.

### Statistical analysis

Data were collected through the MyGut physician dashboard and self-reported study questionnaires and analyzed using GraphPad Prism Software (Version 5; Boston, MA, USA). Comparison of means, such as SIBDQ scores or the number of ER visits/hospitalizations, in the 1 year prior and during the MyGut use, was conducted using a paired *t*-test. Patient satisfaction and feasibility questionnaires were dichotomized into 2 categories: patients who agreed/strongly agreed with the statement and those who were neutral/disagreed/strongly disagreed. Differences between the groups before and after 1 year of MyGut use were analyzed using the McNemar test. *P*-values of <.05 were considered statistically significant. The study participants were included in the final analysis if they completed the baseline and end-of-study questionnaires.

### Ethics statement

This study was approved by the Hamilton Integrated Research Ethics Board and the McGill Research Ethics Board. Written informed consent was obtained from all participants.

## Results

### Participants

Of the 84 patients enrolled in the study, 58 (69%) continued until study completion at 1 year. The median number of questionnaires completed per study participant was 6 (range 0–42). Demographics can be found in Table 1. At baseline, 72.6% of patients (61/84) had already been using technology for health-related purposes. Among those patients, 80.3% (49/61) used technology for researching information about their health, 50.8% (31/61) for health tracking, and 67.2% (41/61) for communicating with their healthcare team (Table 1). There were no significant differences when comparing the demographics of individuals who completed the study vs those who did not (Table S1).

**Table 1.** Patient demographics.

Characteristics	*N* = 84
*Age (years)*	
Mean (SD)	31.03 (9.63)
Median (IQR)	29 (23–37.25)
Min-max	18–60
*Sex, n (%)*	
Male	40 (47.6)
Female	44 (52.4)
*Diagnosis, n (%)*	
Crohn’s disease	58 (69.0)
Ulcerative colitis	25 (29.8)
Other	1 (1.2)
*Smoking status, n (%)*	
Active smoker	10 (11.9)
Past smoker	11 (13.1)
Non-smoker	63 (75.0)
*Do you use technology for health-related purposes? N (%)*	
Yes	61 (72.6)
No	23 (27.4)
*If you use technology for health-related purposes, what do you use it for? N (%)*	
For health tracking	31 (50.8)
For researching health-related information	49 (80.3)
For communicating with healthcare providers	41 (67.2)

### Acceptability

All 84 patients who initially expressed interest in the study agreed to using the application after connecting with the MyGut team following a brief tutorial.

### Satisfaction

No statistically significant differences were observed in the proportion of subjects who strongly agreed/agreed with various statements expressing their satisfaction with care, such as “I am satisfied that I understand the nature and causes of my health condition”, after 1 year of MyGut use when compared with baseline (*P*-values between .48 and 1.0) (Table 2). In a subgroup analysis, the patients who did not continue until study completion were more likely to be satisfied with the education that they received from their healthcare team when compared with those who completed the study (*P* = .001) (Table S2).

**Table 2.** Patient satisfaction at baseline vs after 1 year of MyGut use.

Statement	Number of patients who strongly agree/agree (baseline, *n* = 49)	Number of patients who strongly agree/agree (1 year, *n* = 49)	*P*-value (McNemar test)
I am satisfied with the patient education at McMaster University or McGill University.	37/49	34/49	.61
I am satisfied that my doctor told me about my condition, the treatment options, and how I can stay healthy.	40/49	40/49	1.0
I am satisfied that I understand the nature and causes of my health condition.	35/49	31/49	.48
I am satisfied that I know the different medical treatment options available for my health condition.	38/49	35/49	.63
I am satisfied that I know how to prevent further problems with my health condition.	31/49	28/49	.63

### Quality of life/symptom control

An analysis of SIBDQ scores, serving as a proxy measurement of health-related quality of life and symptom control, revealed a significant improvement in SIBDQ scores after 1 year of MyGut use (mean = 56.0, SD 8.85) when compared with baseline (mean = 52.0, SD 9.84) (*P* = .012).

### Resource utilization

Over the 1-year study period, there was no significant difference in the mean number of ER visits and hospitalizations when comparing the 1 year of MyGut use (36 visits, mean = 0.62, SD = 1.44) with the 1 year prior (40 visits, mean = 0.69, SD = 1.50) (*P* = .78).

### Feasibility

There was a significant decrease in patients who were willing to continue using the application past the study-end point, with only 42.9% (21/49) of patients willing to continue using the application after 1 year, compared with the 71.4% (35/49) who were willing to continue after 2 months (*P* = .001) (Table 3).

**Table 3.** Patient-reported feasibility statements at 8 weeks vs 1 year of MyGut use.

Statement	Number of patients who strongly agree/agree (8 weeks, *n* = 49)	Number of patients who strongly agree/agree (1 year, *n* = 49)	*P*-value (McNemar test)
I am able to effectively learn about my condition.	38/49	30/49	.096
The app increases my access to healthcare services.	22/49	20/49	.803
I believe this app will help me manage my health.	24/49	19/49	.267
It is easy to use and navigate through the app.	42/49	39/49	.581
The app looks pleasant and appealing.	38/49	38/49	1.0
The features within the app are relevant and useful.	34/49	32/49	.791
I have not run into technical difficulties with the app.	40/49	38/49	.688
If I make a mistake in the app, I can easily correct it.	28/49	32/49	.388
I feel comfortable using the app.	42/49	38/49	.125
I would continue using the app.	35/49	21/49	**.001**
Health monitoring apps like MyGut are beneficial for healthcare settings.	39/49	35/49	.388
Overall, I am satisfied with the app.	37/49	31/49	.180

## Discussion

The impact of IBD extends beyond its physical burden and adversely affects patients’ social, professional, and emotional health.^15^ Patient-reported outcomes serve as valuable metrics in understanding patients’ symptoms, quality of life, and satisfaction, and aid in achieving the goal of patient-centred care.^16^ This study demonstrates that patients with IBD are very willing to incorporate digital health monitoring platforms into their healthcare regime, that many patients are already using these health technologies, and that these applications may subsequently lead to improved quality of life. However, while most patients are receptive to digital health monitoring, there is a significant decrease in their willingness to continue using these interventions over time.

The significant increase in mean SIBDQ scores highlights a promising advantage of health monitoring applications. In addition to improvements in quality of life, higher SIBDQ scores have been linked to better disease control and scores above 55 have been associated with mucosal healing.^17^ Furthermore, in contrast to frequent clinic visits that may be time consuming, resource intensive, and low yield, continuous monitoring provided through the physician dashboard can identify patients with suboptimal disease control earlier.^18^ During this study, several patients were contacted due to low SIBDQ scores and had management plans adjusted. While our findings are largely consistent with previous studies on digital health monitoring in IBD, the literature presents mixed results between overall improvements vs no significant changes.^9^ However, most studies are conducted at tertiary care centres where patients receive specialized IBD care with a large multi-disciplinary team, frequent follow-ups, and a high standard of care at baseline.^19^ Accordingly, the benefit of digital health monitoring in these settings may not be as significant. De Garibay et al. conducted a study on mobile health applications for cardiovascular health and found similar rates of health improvements in both rural and urban Spain. This suggests that these tools are also beneficial in resource-limited settings.^20^ Taken together, the collective literature indicates that digital health monitoring applications have the potential to improve quality of life with minimal risks or downsides.

While we noted improvements in SIBDQ scores, there was no significant change in the number of ER visits/hospitalizations following the MyGut use. This is in contrast to a recent study investigating the HealthPROMISE IBD platform which found a significant reduction in the frequency of IBD-related ER visits and hospitalizations.^21^ This difference may arise from the fact that, in our present study, ER visits/hospitalizations were recorded by a small subset of patients with refractory disease. However, other studies have demonstrated a decrease in the number of outpatient visits among their patients using digital health monitoring.^22,23^ Regardless, although our study did not find a significant change in ER visits/hospitalizations, it is possible that, by improving symptom control, we could reduce the need for frequent clinic visits, thereby leading to a reduction in resource utilization without compromising disease control.

Consistent with most other digital health applications, the primary challenge of our study revolved around attrition and overall compliance. While we found that patients are initially very receptive to using digital health monitoring applications, we noted that their willingness to continue drops rapidly over time. With an attrition rate of 31%, our results align with previous eHealth studies, demonstrating that as few as 10%–25% of patients continue entering data by the end of the study and 71% of app users across all industries stop after 3 months.^24,25^ Interestingly, the only difference we found between patients who did not complete the study vs those who did was that patients who left prematurely had higher levels of satisfaction with the education they received from their healthcare team. This may suggest that the patients who felt most confident in managing their condition did not perceive the application as necessary to complement their care.

Factors contributing to attrition in our study include the perceived burden, insufficient understanding of the application’s purpose, and inadequate technical support. The patients who were in remission specifically reported negative feedback regarding the burden of answering the SIBDQ every 2 weeks when their condition remained unchanged. Accordingly, ideal participants may be those with a new diagnosis of IBD, recent ER visits/hospitalizations, recent disease flare, recent changes to their medication regime, or uncontrolled disease at baseline. Studies aimed at maximizing engagement have suggested ensuring patients understand the application’s objectives, reducing the frequency and volume of data collection, shortening the study period, increasing the frequency of reminders, improving technical support, and providing consistent feedback regarding their scores.^24^

Our study’s limitations primarily stem from its single-arm design, its reliance on subjective data, and its relatively small sample size. Due to the lack of a control group, we were unable to compare groups to determine whether improvements in SIBDQ scores may be attributed to MyGut use, a Hawthorne effect, or other changes in the care provided by healthcare teams. Furthermore, although our study used validated measures of symptom control, we were unable to incorporate the use of objective measures, such as inflammatory markers or endoscopic findings, given the lack of infrastructure to implement this into MyGut. Additionally, there may be potential selection bias as participants tended to be younger, were more technologically adept, and possessed a greater proficiency in English, likely owing to the technology-based nature of the study. Furthermore, 72.6% of our participants were already using technology for health-related purposes, compared with 49.2% in a population study of 253 829 participants in the United States. We believe that this discrepancy may be attributable to differences in the study populations, specifically, individuals diagnosed with a chronic disease vs the general population. Lastly, while consistent with other eHealth studies, high attrition rates constrained our ability to conduct comprehensive subgroup analyses. Accordingly, future studies should involve larger randomized controlled trials, using both subjective and objective evidence of disease control, to determine the patient population that would benefit most from these digital health interventions.

## Conclusion

Overall, IBD is a condition that often necessitates frequent medical follow-up and may benefit from proactive symptom management strategies. Digital health monitoring platforms offer a promising avenue to provide more proactive care. MyGut differentiates itself from other IBD applications by incorporating a physician dashboard to facilitate a connection between patients and their healthcare team. Additionally, by integrating validated questionnaires, specifically those encompassing aspects of social/emotional health well-being, healthcare providers gain a more comprehensive understanding of how IBD affects their patients.^26^

This study ultimately demonstrates the high willingness of patients with IBD to integrate digital health monitoring platforms into their care, potentially resulting in improved symptom control and enhanced quality of life. Unfortunately, as with most digital health technologies, continued efforts must be made to optimize the acceptability and feasibility of its long-term use.

## Supplementary data

Supplementary data are available at *Journal of the Canadian Association of Gastroenterology* online.

gwae029_suppl_Supplementary_Tables

gwae029_suppl_Supplementary_Material

## Data Availability

The data underlying this study can be shared upon reasonable request to the corresponding author.
