# Supplementary material for: Acceptability, feasibility, and impact of the MyGut digital health platform in the monitoring and management of inflammatory bowel disease
Source: J Can Assoc Gastroenterol. 2024 Sep 6;7(6):423–30. doi: 10.1093/jcag/gwae029 (PMC11638002; doi:10.1093/jcag/gwae029)
Supplement: gwae029_suppl_Supplementary_Tables [file gwae029_suppl_supplementary_tables.docx]

**Table S1.** Patient demographics among patients who completed the study versus patients who did not

| **Characteristics** | **Patients who completed the study**  **(n = 58)** | **Patients who did not complete the study**  **(n = 26)** | **P-value**  **(chi-square)** |
| --- | --- | --- | --- |
| **Age (years)**  Mean (SD) | 31.03 (9.63) | 30.31 (10.58) | 0.32 |
| **Sex, n (%)**  Male  Female | 40 (47.6)  44 (52.4) | 13 (50.0)  13 (50.0) | 0.77 |
| **Diagnosis, n (%)**  Crohn’s disease  Ulcerative colitis  Other | 58 (69.0)  25 (29.8)  1 (1.2) | 18 (69.2)  8 (30.8)  0 (0.0) | 0.93 |
| **Smoking status, n (%)**  Active smoker  Past smoker  Non-smoker | 10 (11.9)  11 (13.1)  63 (75.0) | 3 (11.5)  5 (19.2)  18 (69.2) | 0.41 |
| **Do you use technology for health-related purposes? N (%)**  Yes  No | 61 (72.6)  23 (27.4) | 18 (69.2)  8 (30.8) | 0.64 |

**Table S2.** Baseline patient satisfaction among patients who completed the study versus patients who did not

| Statement | Number of patients who strongly agree/agree  (Completers, n = 58) | Number of patients who strongly agree/agree  (Non-completers, n = 26) | P value (chi-square) |
| --- | --- | --- | --- |
| I am satisfied with the patient education at McMaster University or McGill University. | 45/58 | 26/26 | **0.001** |
| I am satisfied that my doctor told me about my condition, the treatment options and how I can stay healthy. | 48/58 | 25/26 | 0.09 |
| I am satisfied that I understand the nature and causes of my health condition. | 42/58 | 19/26 | 0.95 |
| I am satisfied that I know the different medical treatment options available for my health condition. | 45/58 | 22/26 | 0.46 |
| I am satisfied that I know how to prevent further problems with my health condition. | 38/58 | 19/26 | 0.49 |

**Table S3.** Feasibility statements at 8 weeks among patients who completed the study versus patients who did not

| Statement | Number of patients who strongly agree/agree  (Completers, n = 51) | Number of patients who strongly agree/agree  (Non-completers, n = 11) | P value (chi-square) |
| --- | --- | --- | --- |
| I am able to effectively learn about my condition. | 39/51 | 9/11 | 0.70 |
| The app increases my access to healthcare services. | 24/51 | 6/11 | 0.65 |
| I believe this app will help me manage my health. | 26/51 | 7/11 | 0.44 |
| It is easy to use and navigate through the app. | 44/51 | 11/11 | 0.19 |
| The app looks pleasant and appealing. | 39/51 | 9/11 | 0.70 |
| The features within the app are relevant and useful. | 36/51 | 10/11 | 0.16 |
| I have not run into technical difficulties with the app. | 42/51 | 9/11 | 0.97 |
| If I make a mistake in the app, I can easily correct it. | 30/51 | 6/11 | 0.79 |
| I feel comfortable using the app. | 44/51 | 11/11 | 0.19 |
| I would continue using the app. | 37/51 | 8/11 | 0.99 |
| Health monitoring apps like MyGut are beneficial for healthcare settings. | 41/51 | 8/11 | 0.57 |
| Overall, I am satisfied with the app. | 39/51 | 9/11 | 0.70 |
